# Attention pyramid pooling network for artificial diagnosis on pulmonary nodules

**DOI:** 10.1371/journal.pone.0302641

**Published:** 2024-05-16

**Authors:** Hongfeng Wang, Hai Zhu, Lihua Ding, Kaili Yang

**Affiliations:** 1 School of Network Engineering, Zhoukou Normal University, Zhoukou, China; 2 College of Public Health, Zhengzhou University, Zhengzhou, China; 3 Henan Provincial People’s Hospital, People’s Hospital of Zhengzhou University, Henan University People’s Hospital, Zhengzhou, China; University of Engineering & Technology, Taxila, PAKISTAN

## Abstract

The development of automated tools using advanced technologies like deep learning holds great promise for improving the accuracy of lung nodule classification in computed tomography (CT) imaging, ultimately reducing lung cancer mortality rates. However, lung nodules can be difficult to detect and classify, from CT images since different imaging modalities may provide varying levels of detail and clarity. Besides, the existing convolutional neural network may struggle to detect nodules that are small or located in difficult-to-detect regions of the lung. Therefore, the attention pyramid pooling network (APPN) is proposed to identify and classify lung nodules. First, a strong feature extractor, named vgg16, is used to obtain features from CT images. Then, the attention primary pyramid module is proposed by combining the attention mechanism and pyramid pooling module, which allows for the fusion of features at different scales and focuses on the most important features for nodule classification. Finally, we use the gated spatial memory technique to decode the general features, which is able to extract more accurate features for classifying lung nodules. The experimental results on the LIDC-IDRI dataset show that the APPN can achieve highly accurate and effective for classifying lung nodules, with sensitivity of 87.59%, specificity of 90.46%, accuracy of 88.47%, positive predictive value of 95.41%, negative predictive value of 76.29% and area under receiver operating characteristic curve of 0.914.

## 1 Introduction

Lung cancer is a very serious disease and is responsible for a large number of cancer-related deaths worldwide [1–4]. Although there have been significant advances in the treatment of lung cancer in recent years, including the development of multi-modality treatments such as surgery, chemotherapy, and radiation therapy, the prognosis for patients with lung cancer remains poor [5]. It is often difficult to detect lung cancer in its early stages, which can lead to poor outcomes for patients. As a result, the 5-year survival rate for lung cancer remains relatively low at 18% [6]. Advanced recognition of lung cancer, using computed tomography (CT) imaging and other diagnostic techniques, can help to identify lung nodules at an early stage and determine whether they are likely to be benign or malignant [7]. This information can help clinicians to make informed decisions about treatment options and improve the chances of successful outcomes [8].

The diagnosis of lung cancer based on CT scans has traditionally relied on the interpretation of radiologists, which is subjective and can be influenced by factors such as fatigue, emotion, and experience [9, 10]. This can lead to variability in the interpretation of CT images and potentially affect the accuracy of lung nodule diagnosis. As the section thickness of CT scans decreases, the ability to distinguish between pulmonary lesions and adjacent normal vascular structures becomes more difficult, particularly for radiologists who rely on their visual interpretation skills to differentiate these structures [11, 12]. Generally, it is difficult for even experienced radiologists to distinguish some benign and malignant nodules with similar visual features [13, 14]. This can lead to a greater chance of false-positive or false-negative diagnoses, as well as a lower overall accuracy of lung nodule classification [15]. Specifically, the automatic classification of malignancy suspiciousness on CT studies can facilitate radiologists to assess early risk factors which is essential in lung cancer research, thereby providing useful cues for subsequent therapeutic planning and holding promise for improving individualized patient management [6, 16, 17]. As a result, there is a need for more objective and quantitative methods for lung nodule classification that could be an important step towards improving patient outcomes and reducing mortality rates [18].

With the increase in computational power, particularly aided by the advent of powerful Graphics Processing Units (GPUs), convolutional neural networks (CNNs) that is a type of deep learning model and is designed to automatically extract features from input data have shown promising results in the classification of pulmonary nodules [19–21]. In the context of pulmonary nodule classification, a CNN is typically trained on large annotated datasets of CT scans of the lung, and learns to identify features that are indicative of nodules [22–24]. Once trained, the network can be used to detect nodules in new, unseen CT scans, with varying levels of success [25–27]. For example, The Nam et al. underscores the potential of DL-based approaches in enhancing the efficiency and accuracy of medical image analysis [28]. By leveraging deep learning techniques, their algorithm achieved remarkable results in both classifying X-ray images based on nodule presence and accurately detecting nodules within the images. Chen et al. [29] utilized multiple CNNs to distinguish between potentially benign, uncertain, and probable malignant lung nodules. [29] method combined the outputs of multiple neural networks to improve the accuracy and reliability of pulmonary nodule classification. Based on their work, [30] proposed a method combining vgg16 and Faster R-CNN for lung nodule detection. Shen et al. [31] proposed a classification method called MCNN, which does not require segmentation of lung nodules. Instead of segmenting individual nodules, MCNN takes as input regions of interest (ROIs) in different sizes and concatenates their response neuron activations in the output layer of the network.

Studies have shown that CNN-based approaches can achieve high levels of sensitivity and specificity in the classification of pulmonary nodules [32, 33]. While CNN-based methodologies have demonstrated significant promise in the categorization of pulmonary nodules, the detection and classification of lung nodules still pose challenges. First, Different imaging modalities may provide varying levels of detail and clarity. Second, CNNs may encounter challenges when attempting to identify nodules inside lung regions that have a high level of noise or artifacts. Additionally, the detection of small nodules or those situated in hard-to-discern areas of the lung may provide further difficulties for CNNs.

In this paper, we proposed attention pyramid pooling network (APPN) appears to be a comprehensive and effective approach to classify lung nodules. The use of a deep neural network, such as vgg16, as an encoder to extract features can help to identify subtle characteristics of the nodules that might not be apparent to the human. The attention primary pyramid module is an important feature of the proposed APPN, as it allows for the fusion of features at different scales and focuses on the most important features for lung nodule classification. Finally, the use of a novel decoder with multiple up-sample operations can help to refine the classification results. These are impressive results on LIDC-IDRI dataset, indicating that the proposed APPN method is highly accurate and effective for classifying lung nodules. The high sensitivity value of 87.59% suggests that the method is able to correctly identify most malignant nodules, while the specificity value of 90.46% indicates that it is also good at distinguishing between benign and malignant nodules. The positive predictive value (PPV) value of 95.41% and negative predictive value (NPV) value of 76.29% further reinforce the accuracy of the method in making correct predictions. These results suggest that the APPN could be a valuable tool for classifying lung nodules in clinical settings, potentially helping to facilitate early diagnosis and improve patient outcomes.

## 2 Materials and methods

### 2.1 Attention pyramid pooling network

**Fig 1:**
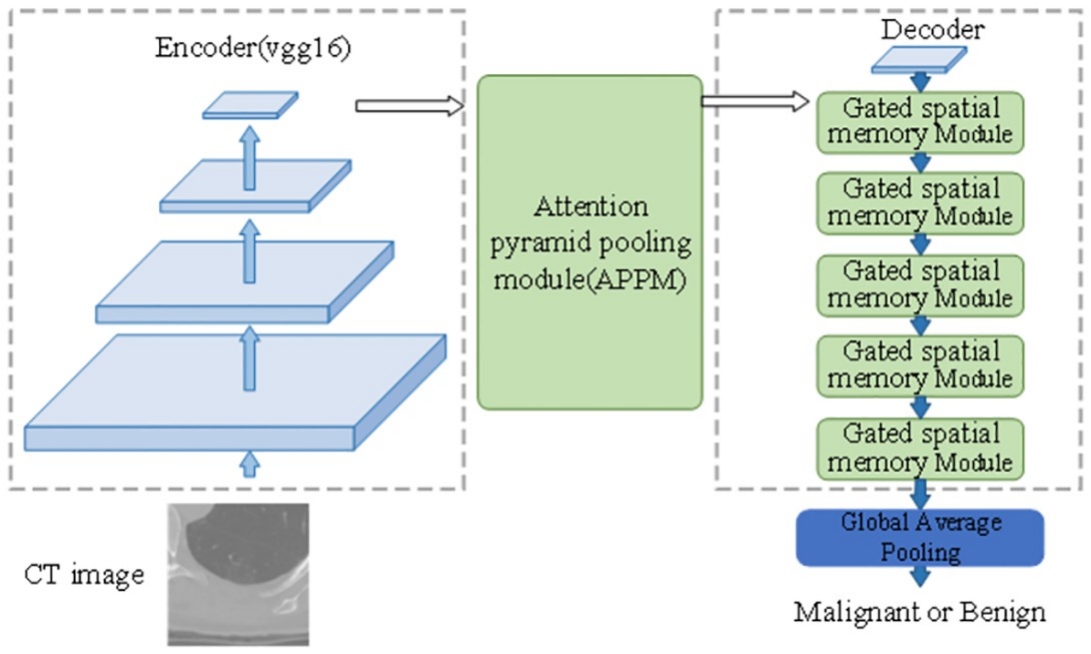
The framework of attention pyramid pooling network.

The APPN follows three steps that will be described below. First, the vgg16 is taken as encoder to extract features of the lung nodules. Then, we propose attention primary pyramid module to fuse features with multiple scales, in which the attention mechanism is used to strengthen the important features. Finally, a novel decoder with multiple up-sample operation is used to classify lung nodules. The proposed methodology is shown in Fig 1.

#### 2.1.1 Encoder

The vgg16 [34, 35] architecture is a 16-layer deep neural network that consists of several convolutional layers, max pooling layers, and fully connected layers. Here is a detail description of each block and its structure, as shown in Table 1.

**Table 1:** The structure of vgg16.

Layer_name	Kernel	Number
Conv1	3×3	64
	3×3	64
Conv2_x	3×3	128
	3×3	128
Conv3_x	3×3	256
	3×3	256
	3×3	256
Conv4_x	3×3	512
	3×3	512
	3×3	512
Conv5_x	3×3	512
	3×3	512
	3×3	512

The first few layers of vgg16 are composed of convolutional layers with small 3×3 filters, followed by a max pooling layer which reduces the spatial dimensions of the output. The last few layers are comprised of fully connected layers that perform a classification task. The final layer of the model is a softmax layer that outputs the predicted probabilities for each class. The vgg16 has a relatively simple architecture compared to some more recent models [36], but it has been shown to be effective for a wide range of computer vision tasks, including image classification, object detection, and segmentation.

#### 2.1.2 Attention pyramid pooling module

**Fig 2:**
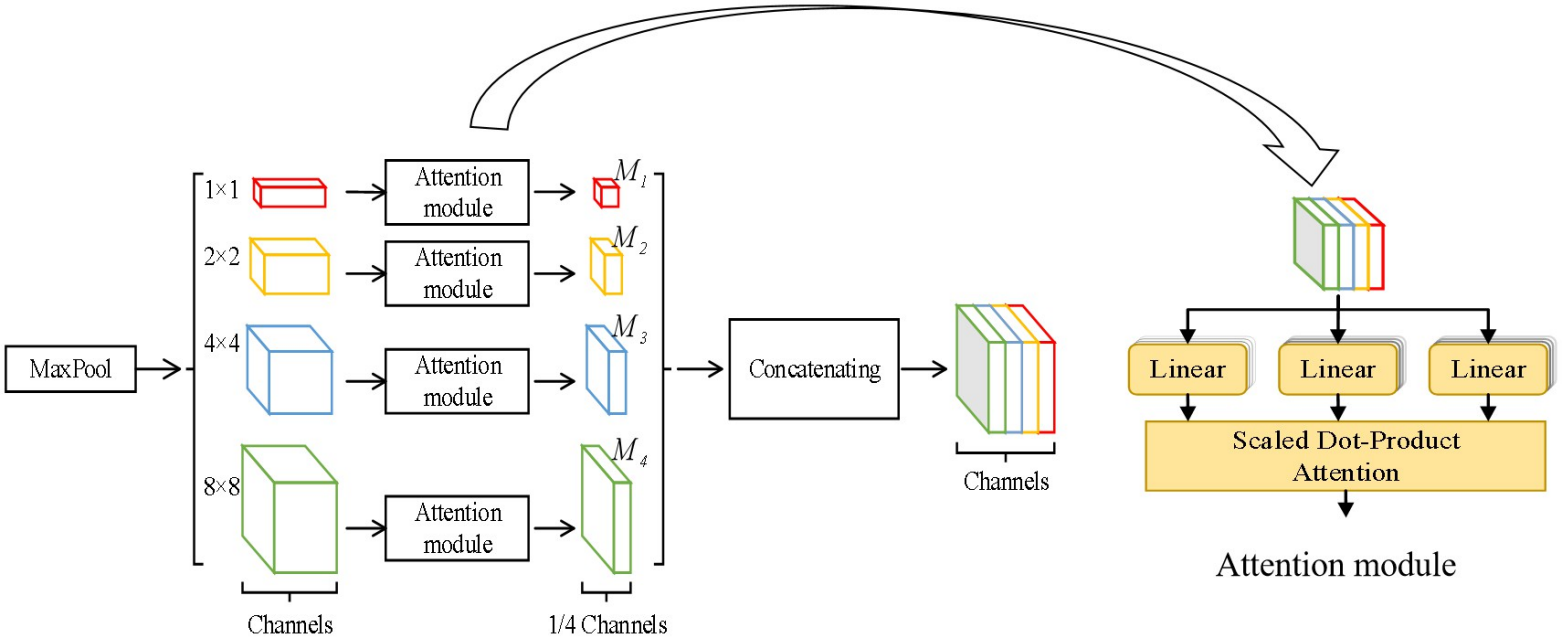
The structure of attention pyramid pooling module.

The pyramid pooling module (PPM) [37–39] is a deep learning architecture used for semantic segmentation tasks in computer vision. The PPM is designed to capture multi-scale contextual information from different regions of an image by dividing the input image into a series of regions or pyramids and pooling features from each region at different scales. However, the PPM is designed to extract the features for object in nature image [37, 40], which is easy to ignore the detail feature. Therefore, we propose attention pyramid pooling module (APPM) by fusing the attention mechanism into the PPM allowing to extract more accurate feature of lung nodule, as shown in Fig 2.

Specially, the input image is fed into a series of convolutional layers to extract features from the image. These layers are typically pre-trained on a large dataset such as ImageNet. Second, four pyramid pooling modules is used to capture multi-scale information from the input image. Each module consists of a set of pooling layers with different pyramid levels (1×1, 2×2, 3×3, and 6×6) to capture different levels of detail in the image. Third, the attention mechanism is embedded to strengthen the important features obtained by different pyramid pooling. Finally, the outputs of these modules are concatenated as the input of decoder.

In proposed APPM, the attention mechanism works by computing a weight for each input element that represents its importance to the task. These weights are then used to compute a weighted sum of the input sequence, where the weights act as a probability distribution over the input elements. The resulting weighted sum is then used as input to the next processing step in the network. As a result, the attention mechanism can help model to focus on certain parts of the input sequence during processing. And it allows the APPM to assign different weights to different parts of the input sequence and selectively process the information that is most relevant to the task.

Overall, the APPM can capture multi-scale information that is the most relevant parts of the lung nodules, which helps to improve the accuracy of lung nodules classification tasks.

#### 2.1.3 Decoder

**Fig 3:**
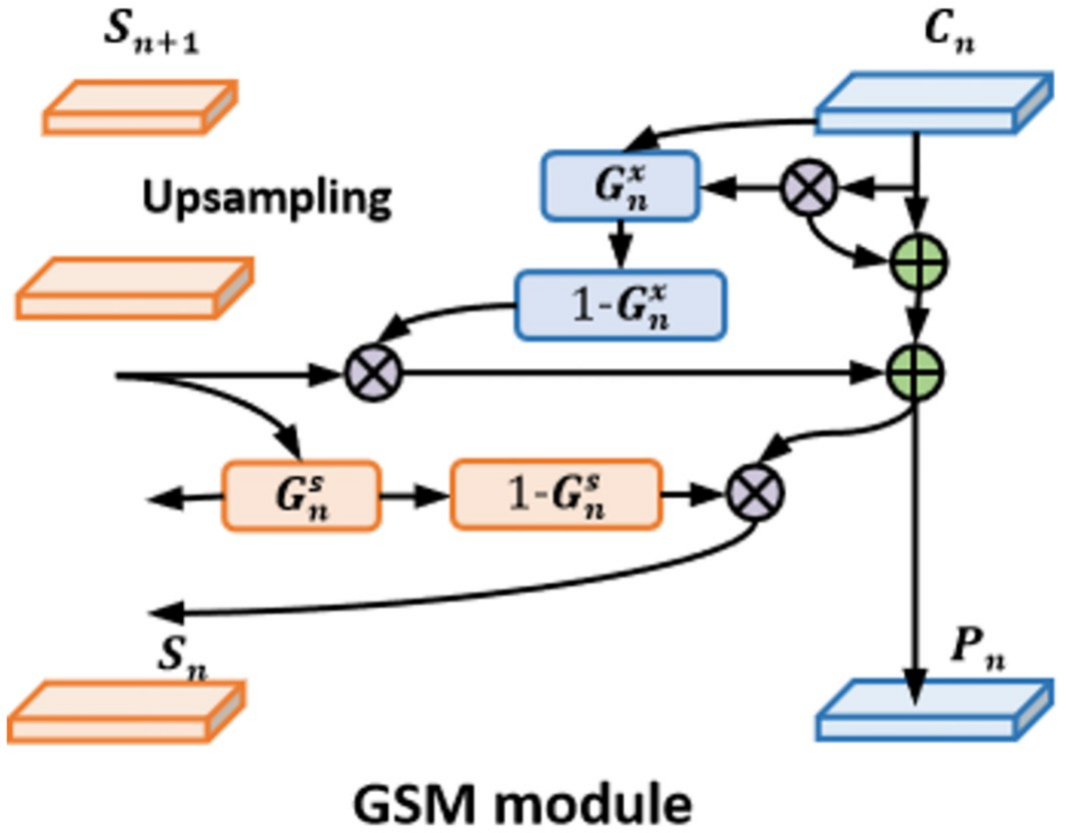
The structure of gated spatial memory.

In decoder, we use gated spatial memory (GSM) technique [41] to decode the general features, which aims to classify the lung nodules. The Structure of GSM is shown in Fig 3. The input of GSM is feature maps generated by APPM.

The appearances of lung nodules always vary greatly in CT images, making the accurate mask prediction challenging. In GSM, a state feature map, such as *S*_*n*_ and *S*_(*n*+1)_, is designed to record the important spatial structure and semantic information. The *G*_*n*_ is the gate layer that is used to update and regulate state feature map. The high gate values in gate layer would strengthen the important feature and the low gate values indicate that the feature is unnecessary. In this way, the state feature map can be used to replenish the lacking information in the feature map of *C*_*n*_.

Finally, the global average pooling is used to aggregate features and the softmax is used to produce the classification scores. Global average pooling has several advantages: (1) the global average pooling reduces the number of parameters in the model, since it requires no learnable parameters. (2) it is less prone to overfitting than max pooling, which tends to preserve spatial information that can be specific to the training set.

### 2.2 Training CNN models

The cross-entropy loss function is utilized to learn the proposed network in order to optimize network parameters for lung nodule classification. The proposed method is implemented using the PyTorch deep learning platform. The influence of learning rate is analyzed through different experiments, and the batch size is also examined in the experiment section. A dropout rate of 0.3 is used during training. All training is conducted on the PyTorch platform, and a NVIDIA GeForce 1080TI graphics processing unit is used to accelerate the training. These details can provide useful information for replicating the proposed method, and the use of open-source tools such as PyTorch can increase the accessibility and reproducibility of the work.

### 2.3 Performance evaluation and statistical analysis

After training the APPN models, the performance is evaluated using the testing dataset, including accuracy, sensitivity, PPV, NPV and area under curve (AUC). The accuracy metric represents the number of correctly classified instances divided by the total number of instances. It gives an overall measure of how well the model is performing. The sensitivity is a metric used to evaluate the performance of a binary classification model, particularly in medical and diagnostic settings. It measures the proportion of positive instances that are correctly identified by the model. The PPV represents the proportion of true positive instances among the instances predicted as positive by the model. The NPV, on the other hand, represents the proportion of true negative instances among the instances predicted as negative by the model. The formulas for accuracy, sensitivity, PPV and NPV are as follows.


$$\text{Accuracy}=\frac{TP+FN}{TP+TN+FP+FN}\times 100\%$$ (1)

$$\text{Sensitivity}=\frac{TP}{TP+FN}\times 100\%$$ (2)

$$\text{PPV}=\frac{TP}{TP+FP}\times 100\%$$ (3)

$$\text{NPV}=\frac{TN}{TN+FN}\times 100\%$$ (4)

True positive (*TP*) represents true positive (the number of malignant nodules that are predicted as malignant nodules). True negative (*TN*) represents true negative (the number of benign nodules that are predicted as benign nodules). False positive (*FP*) represents false positive (the number of benign nodules that are predicted as malignant nodules). False negative (*FN*) represents false negative (the number of malignant nodules that are predicted as benign nodules).

## 3 Experimental results

### 3.1 Dataset preprocessing and splitting

The LIDC-IDRI dataset is a large publicly available dataset of lung CT, it has a total of 1018 cases and each case includes several CT images containing annotated lesions from lung cancer patients. The CT scans in the dataset are available in the DICOM format. The annotation files in the LIDC-IDRI dataset contain information about the location and size of the lesions, as well as their malignancy rating. These labels are extracted using third-party parsing tools (https://github.com/mikejhuang/ LungNoduleDetectionClassification) to fit the needs of the classification task. We denote the dataset that consists of all CT images and labels as lung_all. Meanwhile, to further verify the generalization of the proposed method, we divide the lung_all into lung_1V5 and lung_2V4 datasets according to the malignancy rating. The number of CT scans of lung_all, lung_1V5 and lung_2V4 is shown in Table 2.

**Table 2:** The description of the datasets.

		Training	Testing
lung_all	Overall	5632	1917
	Benign	2041	696
	Malignant	3591	1221
lung_1V5	Overall	2816	953
	Benign	933	313
	Malignant	1883	640
lung_2V4	Overall	2816	964
	Benign	1108	383
	Malignant	1708	581

### 3.2 Parameter analysis

**Fig 4:**
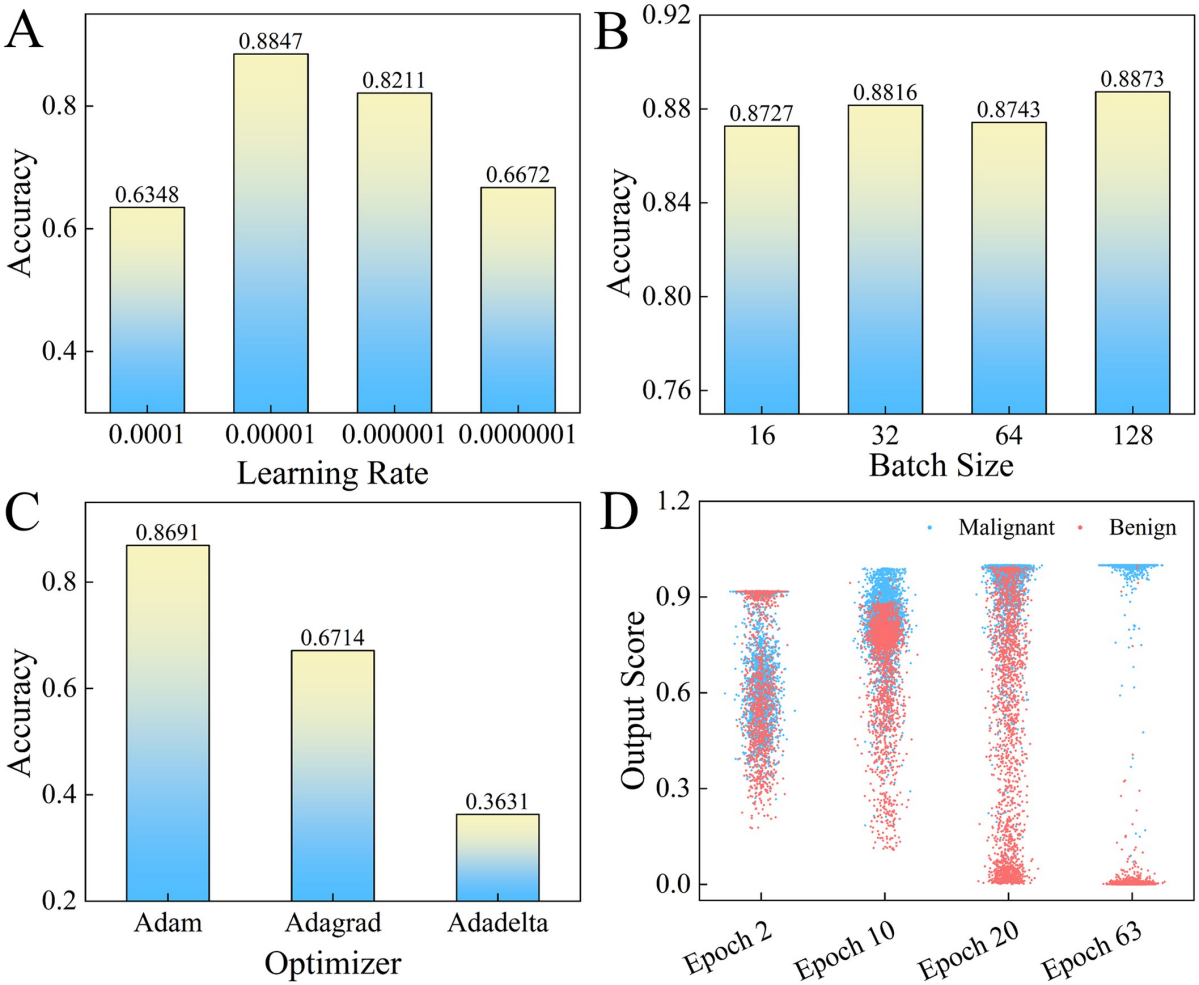
The parameter analysis of learning rate (A), Batch size (B) and optimizer (C) on lung_all. (D) The output scores of training data with the training iterations.

This section describes a study on optimizing the performance of a deep neural network using different hyperparameters, namely, the learning rate, batch size, and optimizer. The study evaluates the performance of the proposed approach with various learning rates of 1e-4, 1e-5, 1e-6, and 1e-7 on lung_all, as shown in Fig 4. The results show that the maximum accuracy (0.8847) is achieved at a learning rate of 1e-5. The impact of batch size on the performance of the network is also investigated, and a range of batch sizes is tested. The results demonstrate that a batch size of 128 obtains the best performance (0.8873) compared to other batch sizes tested. Lastly, the study evaluates the impact of optimizer on the network performance and find that the Adam optimizer achieves the highest accuracy (0.8691). Based on these findings, the optimal hyperparameters for the proposed approach on lung_all are determined to be a learning rate of 1e-5, a batch size of 128, and Adam optimizer. Besides, the final subfigure shows the score tendency of APPN with the increase of epochs. It can be seen that the malignant and benign nodules can be separated gradually when the training epochs increase. These findings highlight the importance of selecting appropriate hyperparameters for deep neural networks when optimizing their performance.

### 3.3 Performance evaluation with deep learning

In this section, we report the performance of APPN on lung_all, such as confusion matrix, sensitivity, specificity, accuracy, PPV, NPV and AUC.

**Fig 5:**
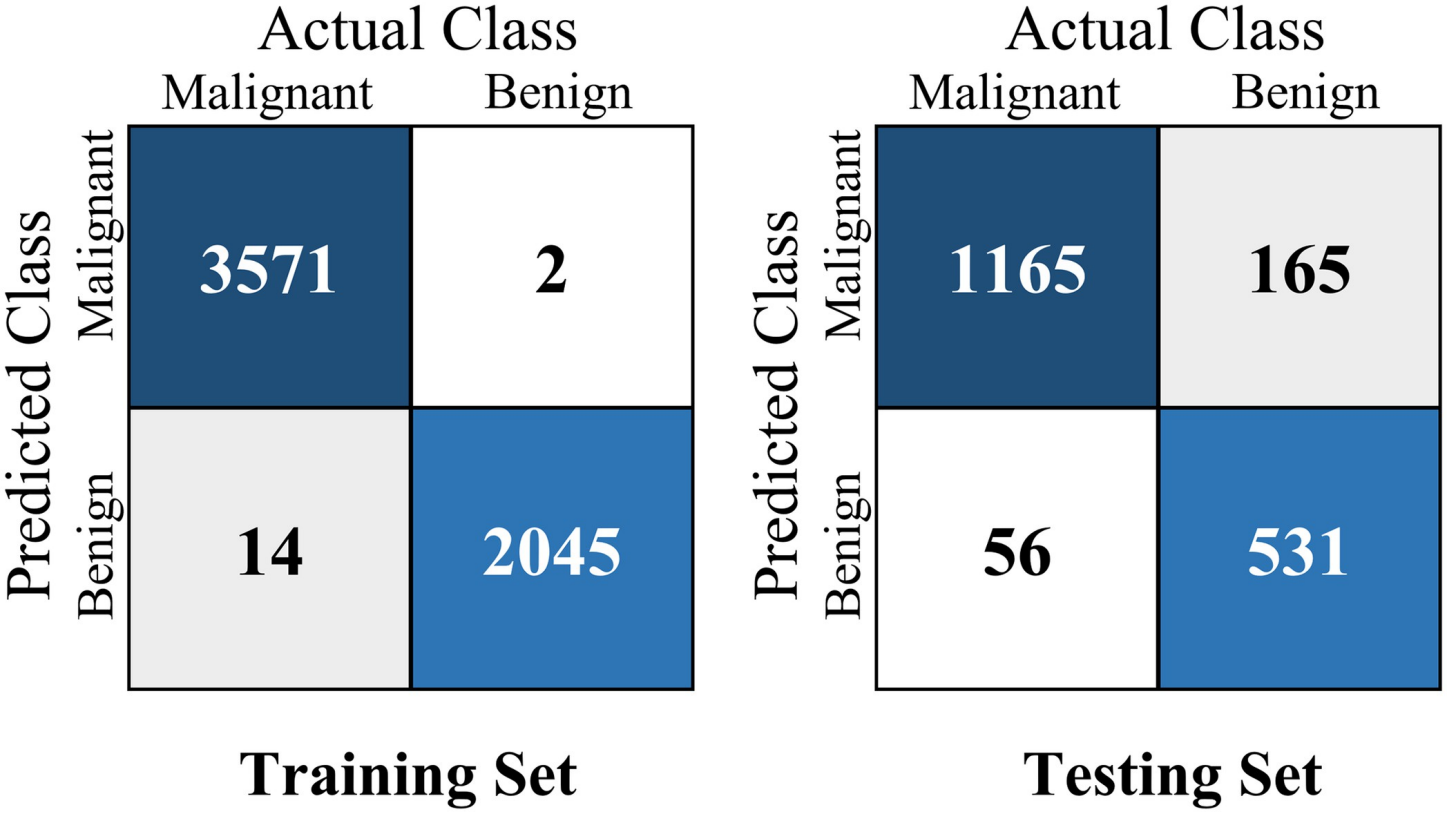
The confusion matrix of the APPN on lung_all.

The confusion matrix provides valuable information about the performance of the proposed method, allowing researchers and practitioners to evaluate the accuracy and effectiveness of the model in classifying lung nodules. The results of confusion matrix are presented in Fig 5. We can see that the APPN performs well on both the training and testing datasets, correctly classifying a significant number of malignant and benign nodules. The confusion matrix shows that 3571 malignant lung nodules and 2045 benign lung nodules are correctly classified by the proposed method on training dataset. And there are 1165 malignant and 531 benign correctly classified by APPN on testing dataset. However, there are some misclassifications on testing dataset, with 165 benign nodules being incorrectly classified as malignant and 56 malignant nodules being misclassified as benign. The numbers from the confusion matrix provide insights into the APPN, helping to evaluate its performance on both the seen (training) and unseen (testing) data.

Table 3 shows the experimental results of APPN on lung_all. The results indicate the satisfied performance of the APPN on the lung_all dataset, both on the training and testing datasets. On the training dataset, the APPN demonstrates exceptional performance with high sensitivity, specificity, and accuracy, indicating its ability to correctly identify both malignant and benign lung nodules. Specially, on the training dataset, the APPN achieves 99.94%, 99.32% and 99.72% in sensitivity, specificity and accuracy, respectively. On the testing dataset, while the model maintains good specificity and accuracy, there is a noticeable decrease in sensitivity. This suggests that the model may be somewhat less effective in identifying true positives on new data. For the testing dataset, the Table 3 shows that the sensitivity, specificity, and accuracy obtained by APPN is 87.59%, 90.46%, 88.47%, respectively. The APPN achieves a PPV of 99.61% and an NPV of 99.90% on the training dataset and a PPV of 95.41% and an NPV of 76.29% on the testing dataset. The PPV on the testing dataset is relatively high (95.41%), indicating that when the model predicts a nodule as malignant, it is correct in a significant majority of cases. It’s essential to consider the specific requirements of the medical application and the implications of false positives and false negatives. Adjustments to the model or further analysis may be necessary depending on the clinical context.

**Table 3:** Experimental results of the APPN on lung_all.

	Training set	Testing set
Sensitivity (%)	99.94	87.59
Specificity (%)	99.32	90.46
Accuracy (%)	99.72	88.47
PPV (%)	99.61	95.41
NPV (%)	99.90	76.29

**Fig 6:**
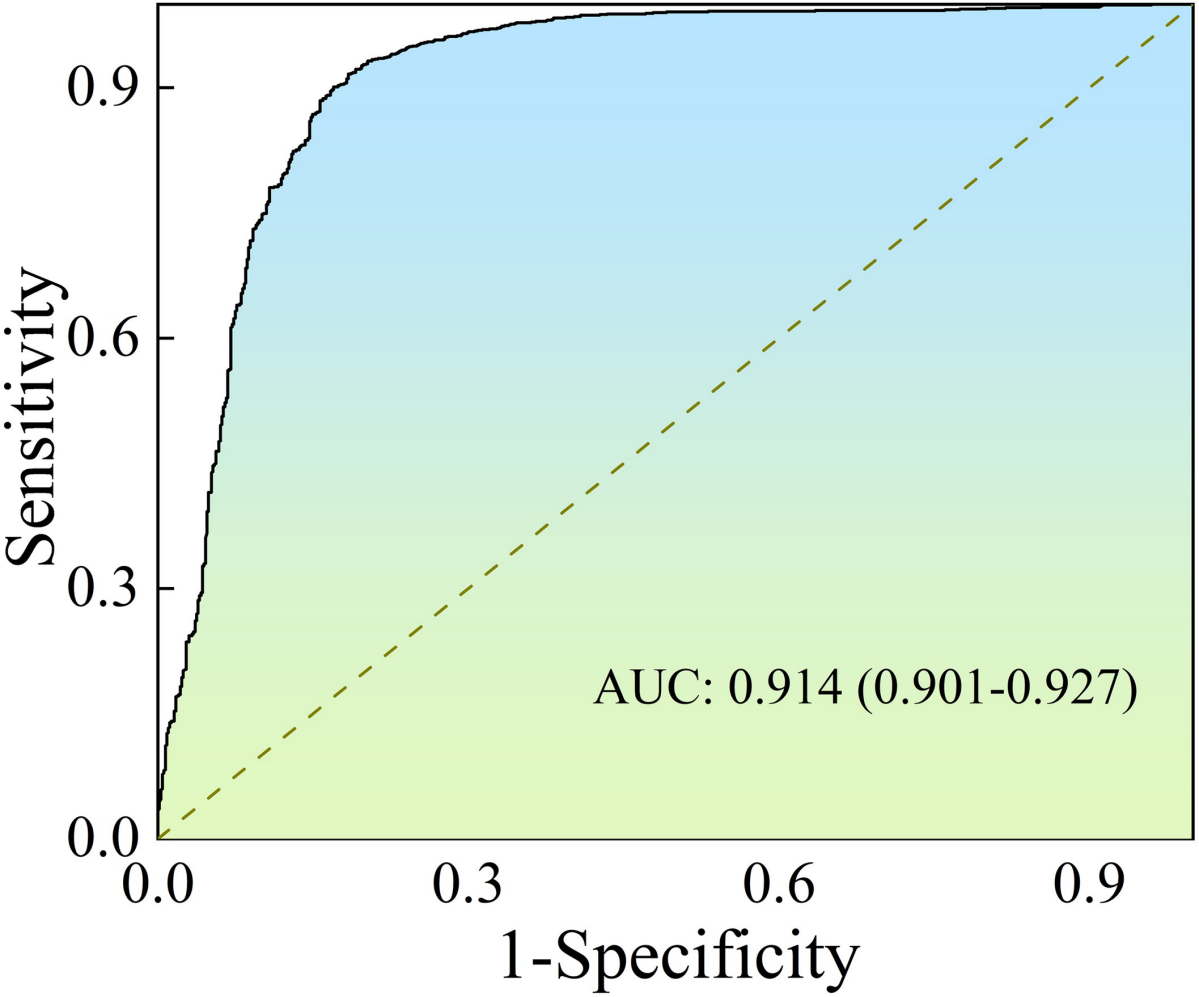
The ROC of the APPN on testing set.

Furthermore, the Fig 6 shows the evaluation of the APPN using the AUC performance metric and the receiver operating characteristic curve (ROC) on lung_all. The APPN achieves an AUC of 0.914 on the testing set. An AUC value close to 1 indicates good discriminatory power of the model. Additionally, the mean time taken for classifying CT images in the testing dataset is around 0.1 seconds. This suggests that the APPN is computationally efficient, demonstrating the ability to handle large datasets in a relatively short amount of time. This efficiency is crucial, especially in medical applications where quick and accurate diagnosis is essential.

In following experimental setup, we replace vgg16 encoder with ResNet18 and ResNet34 for APPN to extract general features from CT images. The results are then compared with the performance in lung nodule classification. The experimental results reported in Table 4 indicates that APPN with vgg16 achieves the best accuracy (88.47%) compared to ResNet18 and ResNet34 encoder. This suggests that, for the specific task of lung nodule classification, vgg16 may be more effective in extracting relevant features from the input data. Besides, ResNet18 and ResNet34 exhibits unsatisfactory accuracy in lung nodule classification. Based on the above analysis, vgg16 is selected as the encoder in the APPN. The reason for this choice is that vgg16 performs better for the lung nodule classification task.

**Table 4:** The performance comparison of vgg16, ResNet18 and ResNet34.

	ResNet18	ResNet34	vgg16
Sensitivity (%)	75.13	73.26	87.59
Specificity (%)	86.27	84.06	90.46
Accuracy (%)	77.20	75.07	88.47
PPV (%)	95.99	95.82	95.41
NPV (%)	44.25	38.65	76.29

In Table 5, the performance of APPN on the LIDC-IDRI is reported alongside other classification methods. The results indicate that APPN achieves the best performance among the compared methods. Benefits from the attention module and PPM, the APPN shows the significant improvements compared to the baseline Autoencoder. The accuracy of APPN is improved by 8.18% compared with the Autoencoder. And the AUC of APPN achieves 0.914 and increases by 5.4% than Autoencoder. Additionally, the APPN shows consider improvement than the second best method which only achieves 87.79 in accuracy. These experimental results validate the effectiveness and superiority of the APPN in lung nodules classification.

**Table 5:** Performance of the proposed method with different methods.

Method	Accuracy (%)	AUC	Ref
Autoencoder	80.29	0.86	[6]
Massive-feat	83.21	0.89	
DGMM-RBCNN	87.79	-	[10]
Hybrid CNN	82.2	0.877	[42]
Resnet152	86	0.84	[43]
Senet154	87	0.84	[44]
APPN	88.47	0.914	This work

### 3.4 Generalization evaluation of the APPN on the lung_1V5 and lung_2V4

In the LIDC-IDRI dataset, the nodules are labelled according to their malignancy levels by four radiologists. The level 1 and 2 represent benign nodules while levels 4 and 5 are malignant nodules. To further verify the generalization of the APPN, the experiments on lung_1V5 and lung_2V4 are conducted from parameter analysis and performance analysis. The malignancy rating with 1 and 5 levels consists of lung_1V5. The malignancy rating with 2 and 4 levels consists of lung_2V4. The number of CT scans of lung_1V5 and lung_2V4 is shown in Table 2.

#### 3.4.1 Experimental results on lung_1V5

The lung_1V5 is build based on malignancy rating with 1 and 5 levels. This dataset has relatively small training sample compared with lung_all. Therefore, it is more difficult to achieve satisfied performance on lung_1V5. And the training dataset of lung_1V5 includes 933 benign and 1883 malignant lunge nodules. The testing dataset of lung_1V5 includes 313 benign and 640 malignant lunge nodules.

**Fig 7:**
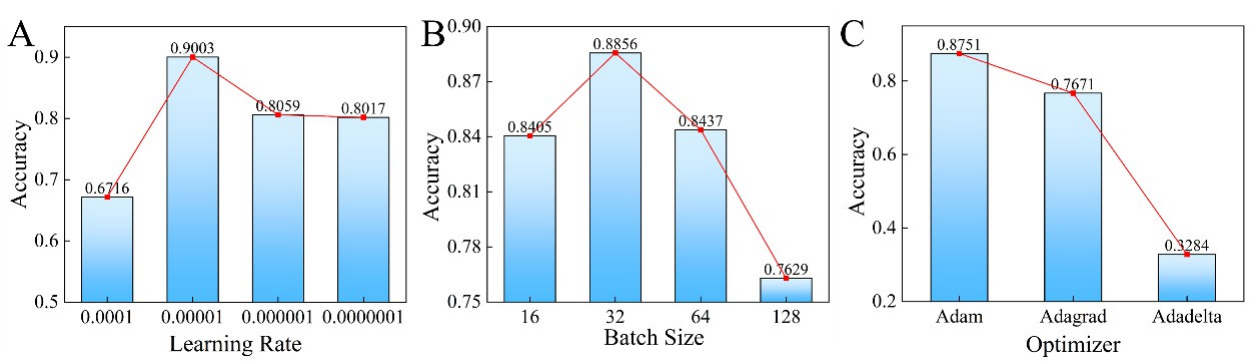
Parameter analysis of learning rate (A), batch size (B) and optimizer (C) on lung_1V5.

We first conduct the parameters analysis experiments on lung_1V5. The experimental results are reported by Fig 7. It is clearly that the APPN can achieve the best accuracy (0.9003) when learning rate is 1e-5. And the 0.8856 accuracy is obtained with 32 batch size. The APPN trained with Adam optimizer achieves the best accuracy (0.8751), which significantly outperforms the accuracy obtained by Adagrad, Adadelta optimizer.

Furthermore, the sensitivity, specificity, sensitivity, PPV, NPV are used to evaluate the performance of the APPN. From the information provided in Table 6, it seems that the APPN on the lung_1V5 dataset outperforms the APPN on the lung_all dataset in terms of sensitivity, specificity, PPV, NPV, and overall accuracy. Specifically, it can be seen that the 90.03% accuracy could be obtained by the APPN on testing datasets, respectively, which is higher than accuracy of the APPN trained with the lung_all. Besides, the APPN reaches a better performance of specificity of 95.8%, PPV of 98.44%, NPV of 72.84%. A high training sensitivity, specificity, accuracy PPV and NPV suggests that the model has learned well from the training data. In summary, the experimental results that the proposed APPN method performs well, especially when testing on the lung_1V5 dataset compared to the lung_all dataset. This suggests that the model is capable of achieving excellent classification performance for identifying lung nodules.

**Table 6:** Experimental results of the APPN on the lung_1V5.

	Testing set
Sensitivity (%)	88.11
Specificity (%)	95.80
Accuracy (%)	90.03
PPV (%)	98.44
NPV (%)	72.84

#### 3.4.2 Experimental results on lung_2V4

The lung_2V4 presents a challenge since some nodules may be difficult to detect or differentiate from other features in the lungs. Besides, the lung_2V4 only contains 2816 training samples, including 1108 benign and 1708 malignant lunge nodules, which is relatively small compared to lung_all. The testing dataset of lung_2V4 includes 383 benign and 581 malignant lunge nodules. Overall, this make it an interesting benchmark for evaluating the performance of lung nodule classification algorithms.

**Fig 8:**
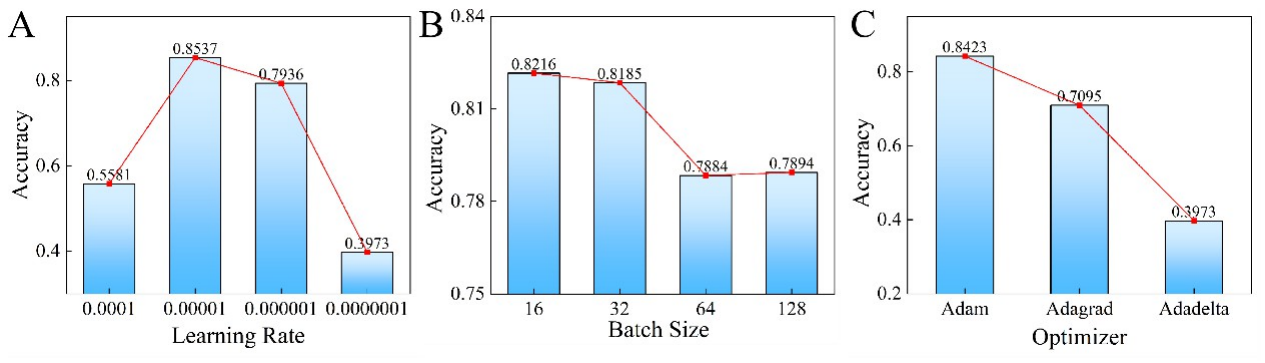
Parameters analysis of learning rate (A), batch size (B) and optimizer (C) on lung_2V4.

First, we conduct experiments to analyze parameter influence of APPN on the lung_2V4, as shown in Fig 8. It can be seen that the APPN is able to achieve the highest accuracy 0.8537 at a learning rate of 1e-5. The experiment also shows that a batch size of 16 resulted in an accuracy of 0.8216. Additionally, the APPN algorithm trained with the Adam optimizer achieves the highest accuracy of 0.8423, surpassing other optimizer used in the experiment.

The information from Table 7 indicates that the APPN is tested on the lung_2V4 dataset, which contains CT images with confusing lung nodules. Since the lung_2V4 contained the CT image with confusion lung nodules, the APPN shows relatively low accuracy on the testing dataset of the lung_2V4. The sensitivity metric indicates the proportion of actual positive cases correctly identified by the model. In this case, it’s 83.72%, suggesting that the APPN successfully classifies a significant portion of the true positive cases, but there is room for improvement. The specificity measures the proportion of actual negative cases correctly identified by the model, and it’s 85.85%. The overall accuracy of 84.44% reflects the proportion of correct predictions (both true positives and true negatives) out of the total instances. The PPV and NPV obtained by APPN only achieves 92.08%, and 72.85% on the testing dataset. The results suggest that the APPN algorithm performs reasonably. It is worth noting that the lung_2V4 dataset is known to be challenging due to the presence of confusing nodules, and therefore achieving high accuracy on this dataset is not a trivial task. These performance metrics suggest that the APPN algorithm is able to achieve relatively high accuracy in classifying lung nodules, but with some room for improvement in terms of sensitivity and accuracy.

**Table 7:** Experimental results of the APPN on the lung_2V4.

	Testing set
Sensitivity (%)	83.72
Specificity (%)	85.85
Accuracy (%)	84.44
PPV (%)	92.08
NPV (%)	72.85

## 4 Discussion

The proposed method, APPN, which is designed based on vgg16 and PPM, with the aim of achieving high accuracy in the classification of lung nodules. Besides, the attention mechanism is adapted to improve the ability capturing the fine feature of lung nodules. Overall, APPN is designed to enhance the ability of the model to extract general features from the lung nodules while also capturing the contextual features in multiple scales, ultimately leading to improved performance in the classification task.

First, we report the parameter analysis of the APPN on lung_all in section 3.2. From the experimental results, it seems that the performance of the APPN algorithm is sensitive to the choice of learning rate and batch size. Specifically, the APPN algorithm achieves the better accuracy of 0.8847 when using learning rate of 1e-5. It is interesting to note that our method with a batch size of 128 achieves the best performance and surpasses the other batch size values significantly. This result suggests that using a batch size of 128 may be optimal for our particular problem and model architecture. However, it is important to note that the optimal batch size may vary depending on various factors such as the size of the dataset, the complexity of the model, and the available computational resources. These results experiments suggest that careful tuning of these hyperparameters can result in significant improvement in classification performance. Additionally, the APPN algorithm trained with Adam achieves a better accuracy (0.8691). This result suggests that the choice of optimizer can also have a significant impact on the performance of the algorithm.

Second, the massive experiments on lung_all are conducted in Second 3.3 for evaluating the performance of the APPN. These performance metrics provide additional information on the precision and accuracy of the proposed method in classifying lung nodules. The high PPV indicates that the proposed method has a low rate of false positives, while the high NPV indicates that the proposed method has a low rate of false negatives. These metrics, along with the previously mentioned metrics (sensitivity, specificity, and accuracy), give a comprehensive evaluation of the performance of the proposed method. Overall, these results suggest that the APPN shows good performance in classifying lung nodules, achieving strong results on the training dataset and relatively accurate results on the testing dataset. However, it should be noted that misclassifications are still present in the testing set, indicating that there is room for improvement in the proposed method. These findings will be helpful in optimizing the proposed method in future iterations, allowing for better classification of lung nodules.

Third, in order to verify the generalization of APPN, we conduct experiments on lung_1V5 and lung_2V4 in Section 3.4. Since these datasets have less training samples than lung_all, they are more difficult to achieve satisfied performance. Specially, a number of confusion lung nodules are contained in lung_2V4 and thus it is an interesting benchmark for evaluating the performance of lung nodule classification algorithms. However, the APPN obtains the good result mainly because the multiple scales contextual features may contribute to obtain the shape and the texture of multiple different dimensions. In APPN, attention mechanism is introduced to obtain the features of small features, so the feature extractor compared with convolutional operation has strong ability. The APPN has a better effect on accuracy of 90.03% on lung_1V5 and accuracy of 84.44% lung_2V4, indicating the proposed model has great application prospects in the early diagnosis of pulmonary nodules.

## 5 Conclusion

In conclusion, one novel deep neural networks, named APPN, is proposed to classify malignant and benign lung nodules based on CT images. The LIDC-IDRI dataset, which is a publicly available dataset of lung CT scans, is used for the development and evaluation of APPN for the classification of pulmonary nodules. The experimental results suggest that the APPN achieves better performance with 88.73% accuracy on the LIDC-IDRI. Besides, the reported accuracy of 90.03% and 84.44% on the lung_1V5 and lung_2V4 suggests that the APPN is effective in accurately predicting the presence of pulmonary nodules in the LIDC-IDRI, which can be expected to improve accuracy of the other database. The APPN can be generalized to the design of high-performance for other medical imaging tasks in the future.

## Supporting information

S1 Dataset (ZIP)
