# Supplementary figures and images for: Attention pyramid pooling network for artificial diagnosis on pulmonary nodules (part 2 of 2)
Source: PLoS One. 2024 May 16;19(5):e0302641. doi: 10.1371/journal.pone.0302641 (PMC11098435; doi:10.1371/journal.pone.0302641)

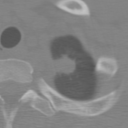

Supplement: S1 Dataset — (ZIP) [file pone.0302641.s001.zip › minimal-dataset/image/0015_NI000_slice014.png]

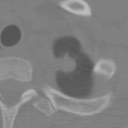

Supplement: S1 Dataset — (ZIP) [file pone.0302641.s001.zip › minimal-dataset/image/0015_NI000_slice015.png]

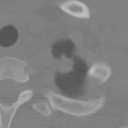

Supplement: S1 Dataset — (ZIP) [file pone.0302641.s001.zip › minimal-dataset/image/0015_NI000_slice016.png]

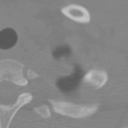

Supplement: S1 Dataset — (ZIP) [file pone.0302641.s001.zip › minimal-dataset/image/0015_NI000_slice017.png]

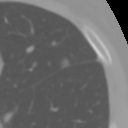

Supplement: S1 Dataset — (ZIP) [file pone.0302641.s001.zip › minimal-dataset/image/0016_NI000_slice000.png]

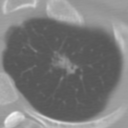

Supplement: S1 Dataset — (ZIP) [file pone.0302641.s001.zip › minimal-dataset/image/0016_NI005_slice002.png]

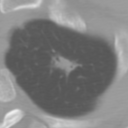

Supplement: S1 Dataset — (ZIP) [file pone.0302641.s001.zip › minimal-dataset/image/0016_NI005_slice003.png]

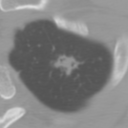

Supplement: S1 Dataset — (ZIP) [file pone.0302641.s001.zip › minimal-dataset/image/0016_NI005_slice004.png]

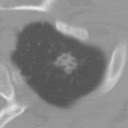

Supplement: S1 Dataset — (ZIP) [file pone.0302641.s001.zip › minimal-dataset/image/0016_NI005_slice005.png]

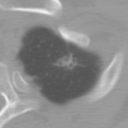

Supplement: S1 Dataset — (ZIP) [file pone.0302641.s001.zip › minimal-dataset/image/0016_NI005_slice006.png]

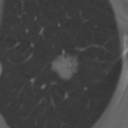

Supplement: S1 Dataset — (ZIP) [file pone.0302641.s001.zip › minimal-dataset/image/0018_NI003_slice001.png]

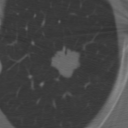

Supplement: S1 Dataset — (ZIP) [file pone.0302641.s001.zip › minimal-dataset/image/0018_NI003_slice002.png]

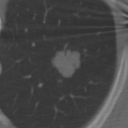

Supplement: S1 Dataset — (ZIP) [file pone.0302641.s001.zip › minimal-dataset/image/0018_NI003_slice003.png]

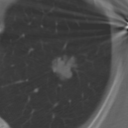

Supplement: S1 Dataset — (ZIP) [file pone.0302641.s001.zip › minimal-dataset/image/0018_NI003_slice004.png]

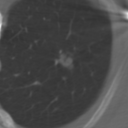

Supplement: S1 Dataset — (ZIP) [file pone.0302641.s001.zip › minimal-dataset/image/0018_NI003_slice005.png]

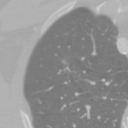

Supplement: S1 Dataset — (ZIP) [file pone.0302641.s001.zip › minimal-dataset/image/0019_NI000_slice000.png]

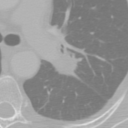

Supplement: S1 Dataset — (ZIP) [file pone.0302641.s001.zip › minimal-dataset/image/0020_NI000_slice001.png]

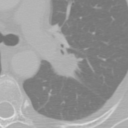

Supplement: S1 Dataset — (ZIP) [file pone.0302641.s001.zip › minimal-dataset/image/0020_NI000_slice002.png]

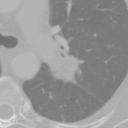

Supplement: S1 Dataset — (ZIP) [file pone.0302641.s001.zip › minimal-dataset/image/0020_NI000_slice003.png]

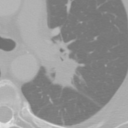

Supplement: S1 Dataset — (ZIP) [file pone.0302641.s001.zip › minimal-dataset/image/0020_NI000_slice004.png]

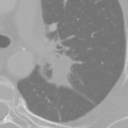

Supplement: S1 Dataset — (ZIP) [file pone.0302641.s001.zip › minimal-dataset/image/0020_NI000_slice005.png]

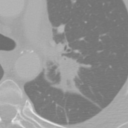

Supplement: S1 Dataset — (ZIP) [file pone.0302641.s001.zip › minimal-dataset/image/0020_NI000_slice006.png]

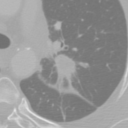

Supplement: S1 Dataset — (ZIP) [file pone.0302641.s001.zip › minimal-dataset/image/0020_NI000_slice007.png]

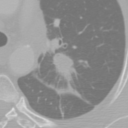

Supplement: S1 Dataset — (ZIP) [file pone.0302641.s001.zip › minimal-dataset/image/0020_NI000_slice008.png]

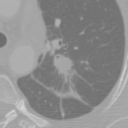

Supplement: S1 Dataset — (ZIP) [file pone.0302641.s001.zip › minimal-dataset/image/0020_NI000_slice009.png]

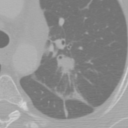

Supplement: S1 Dataset — (ZIP) [file pone.0302641.s001.zip › minimal-dataset/image/0020_NI000_slice010.png]

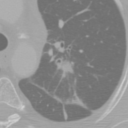

Supplement: S1 Dataset — (ZIP) [file pone.0302641.s001.zip › minimal-dataset/image/0020_NI000_slice011.png]

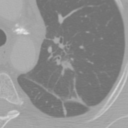

Supplement: S1 Dataset — (ZIP) [file pone.0302641.s001.zip › minimal-dataset/image/0020_NI000_slice012.png]

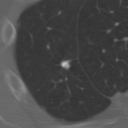

Supplement: S1 Dataset — (ZIP) [file pone.0302641.s001.zip › minimal-dataset/image/0021_NI002_slice002.png]

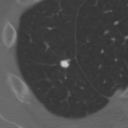

Supplement: S1 Dataset — (ZIP) [file pone.0302641.s001.zip › minimal-dataset/image/0021_NI002_slice003.png]

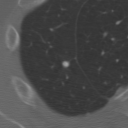

Supplement: S1 Dataset — (ZIP) [file pone.0302641.s001.zip › minimal-dataset/image/0021_NI002_slice004.png]

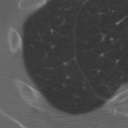

Supplement: S1 Dataset — (ZIP) [file pone.0302641.s001.zip › minimal-dataset/image/0021_NI002_slice005.png]

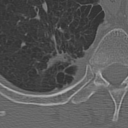

Supplement: S1 Dataset — (ZIP) [file pone.0302641.s001.zip › minimal-dataset/image/0022_NI000_slice000.png]

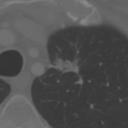

Supplement: S1 Dataset — (ZIP) [file pone.0302641.s001.zip › minimal-dataset/image/0023_NI000_slice002.png]

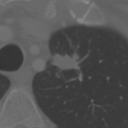

Supplement: S1 Dataset — (ZIP) [file pone.0302641.s001.zip › minimal-dataset/image/0023_NI000_slice003.png]

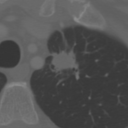

Supplement: S1 Dataset — (ZIP) [file pone.0302641.s001.zip › minimal-dataset/image/0023_NI000_slice004.png]

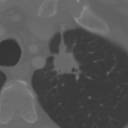

Supplement: S1 Dataset — (ZIP) [file pone.0302641.s001.zip › minimal-dataset/image/0023_NI000_slice005.png]

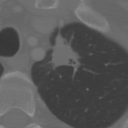

Supplement: S1 Dataset — (ZIP) [file pone.0302641.s001.zip › minimal-dataset/image/0023_NI000_slice006.png]

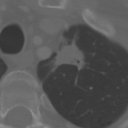

Supplement: S1 Dataset — (ZIP) [file pone.0302641.s001.zip › minimal-dataset/image/0023_NI000_slice007.png]

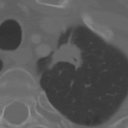

Supplement: S1 Dataset — (ZIP) [file pone.0302641.s001.zip › minimal-dataset/image/0023_NI000_slice008.png]

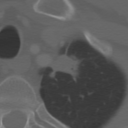

Supplement: S1 Dataset — (ZIP) [file pone.0302641.s001.zip › minimal-dataset/image/0023_NI000_slice009.png]

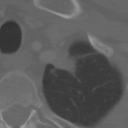

Supplement: S1 Dataset — (ZIP) [file pone.0302641.s001.zip › minimal-dataset/image/0023_NI000_slice010.png]

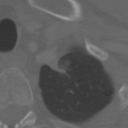

Supplement: S1 Dataset — (ZIP) [file pone.0302641.s001.zip › minimal-dataset/image/0023_NI000_slice011.png]

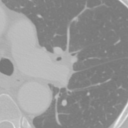

Supplement: S1 Dataset — (ZIP) [file pone.0302641.s001.zip › minimal-dataset/image/0029_NI000_slice001.png]

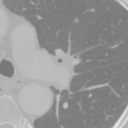

Supplement: S1 Dataset — (ZIP) [file pone.0302641.s001.zip › minimal-dataset/image/0029_NI000_slice002.png]

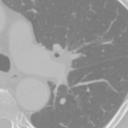

Supplement: S1 Dataset — (ZIP) [file pone.0302641.s001.zip › minimal-dataset/image/0029_NI000_slice003.png]

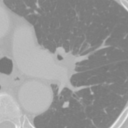

Supplement: S1 Dataset — (ZIP) [file pone.0302641.s001.zip › minimal-dataset/image/0029_NI000_slice004.png]

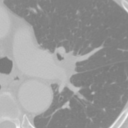

Supplement: S1 Dataset — (ZIP) [file pone.0302641.s001.zip › minimal-dataset/image/0029_NI000_slice005.png]

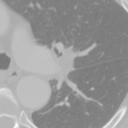

Supplement: S1 Dataset — (ZIP) [file pone.0302641.s001.zip › minimal-dataset/image/0029_NI000_slice006.png]

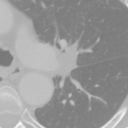

Supplement: S1 Dataset — (ZIP) [file pone.0302641.s001.zip › minimal-dataset/image/0029_NI000_slice007.png]

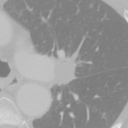

Supplement: S1 Dataset — (ZIP) [file pone.0302641.s001.zip › minimal-dataset/image/0029_NI000_slice008.png]

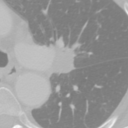

Supplement: S1 Dataset — (ZIP) [file pone.0302641.s001.zip › minimal-dataset/image/0029_NI000_slice009.png]

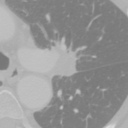

Supplement: S1 Dataset — (ZIP) [file pone.0302641.s001.zip › minimal-dataset/image/0029_NI000_slice010.png]

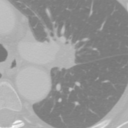

Supplement: S1 Dataset — (ZIP) [file pone.0302641.s001.zip › minimal-dataset/image/0029_NI000_slice011.png]

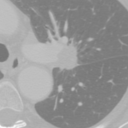

Supplement: S1 Dataset — (ZIP) [file pone.0302641.s001.zip › minimal-dataset/image/0029_NI000_slice012.png]

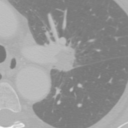

Supplement: S1 Dataset — (ZIP) [file pone.0302641.s001.zip › minimal-dataset/image/0029_NI000_slice013.png]

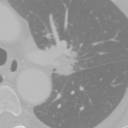

Supplement: S1 Dataset — (ZIP) [file pone.0302641.s001.zip › minimal-dataset/image/0029_NI000_slice014.png]

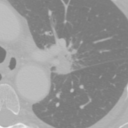

Supplement: S1 Dataset — (ZIP) [file pone.0302641.s001.zip › minimal-dataset/image/0029_NI000_slice015.png]

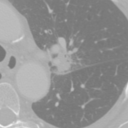

Supplement: S1 Dataset — (ZIP) [file pone.0302641.s001.zip › minimal-dataset/image/0029_NI000_slice016.png]

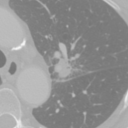

Supplement: S1 Dataset — (ZIP) [file pone.0302641.s001.zip › minimal-dataset/image/0029_NI000_slice017.png]

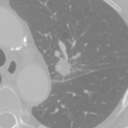

Supplement: S1 Dataset — (ZIP) [file pone.0302641.s001.zip › minimal-dataset/image/0029_NI000_slice018.png]

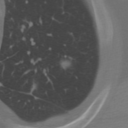

Supplement: S1 Dataset — (ZIP) [file pone.0302641.s001.zip › minimal-dataset/image/0031_NI004_slice002.png]

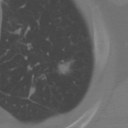

Supplement: S1 Dataset — (ZIP) [file pone.0302641.s001.zip › minimal-dataset/image/0031_NI004_slice003.png]

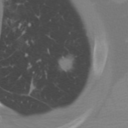

Supplement: S1 Dataset — (ZIP) [file pone.0302641.s001.zip › minimal-dataset/image/0031_NI004_slice004.png]

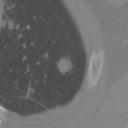

Supplement: S1 Dataset — (ZIP) [file pone.0302641.s001.zip › minimal-dataset/image/0031_NI004_slice005.png]

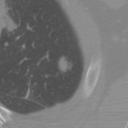

Supplement: S1 Dataset — (ZIP) [file pone.0302641.s001.zip › minimal-dataset/image/0031_NI004_slice006.png]

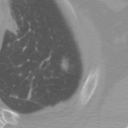

Supplement: S1 Dataset — (ZIP) [file pone.0302641.s001.zip › minimal-dataset/image/0031_NI004_slice007.png]

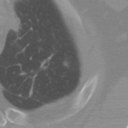

Supplement: S1 Dataset — (ZIP) [file pone.0302641.s001.zip › minimal-dataset/image/0031_NI004_slice008.png]

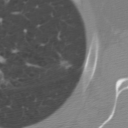

Supplement: S1 Dataset — (ZIP) [file pone.0302641.s001.zip › minimal-dataset/image/0033_NI000_slice000.png]

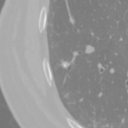

Supplement: S1 Dataset — (ZIP) [file pone.0302641.s001.zip › minimal-dataset/image/0039_NI000_slice001.png]

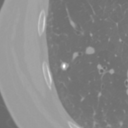

Supplement: S1 Dataset — (ZIP) [file pone.0302641.s001.zip › minimal-dataset/image/0039_NI000_slice002.png]

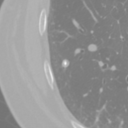

Supplement: S1 Dataset — (ZIP) [file pone.0302641.s001.zip › minimal-dataset/image/0039_NI000_slice003.png]

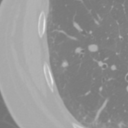

Supplement: S1 Dataset — (ZIP) [file pone.0302641.s001.zip › minimal-dataset/image/0039_NI000_slice004.png]

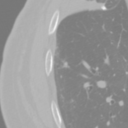

Supplement: S1 Dataset — (ZIP) [file pone.0302641.s001.zip › minimal-dataset/image/0039_NI001_slice001.png]

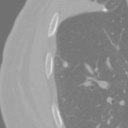

Supplement: S1 Dataset — (ZIP) [file pone.0302641.s001.zip › minimal-dataset/image/0039_NI001_slice002.png]

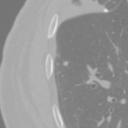

Supplement: S1 Dataset — (ZIP) [file pone.0302641.s001.zip › minimal-dataset/image/0039_NI001_slice003.png]

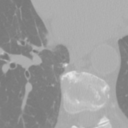

Supplement: S1 Dataset — (ZIP) [file pone.0302641.s001.zip › minimal-dataset/image/0039_NI002_slice001.png]

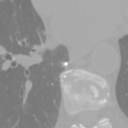

Supplement: S1 Dataset — (ZIP) [file pone.0302641.s001.zip › minimal-dataset/image/0039_NI002_slice002.png]

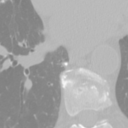

Supplement: S1 Dataset — (ZIP) [file pone.0302641.s001.zip › minimal-dataset/image/0039_NI002_slice003.png]

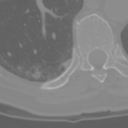

Supplement: S1 Dataset — (ZIP) [file pone.0302641.s001.zip › minimal-dataset/image/0039_NI003_slice000.png]

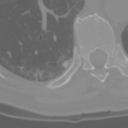

Supplement: S1 Dataset — (ZIP) [file pone.0302641.s001.zip › minimal-dataset/image/0039_NI003_slice001.png]

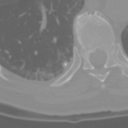

Supplement: S1 Dataset — (ZIP) [file pone.0302641.s001.zip › minimal-dataset/image/0039_NI003_slice002.png]

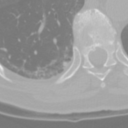

Supplement: S1 Dataset — (ZIP) [file pone.0302641.s001.zip › minimal-dataset/image/0039_NI003_slice003.png]

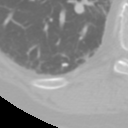

Supplement: S1 Dataset — (ZIP) [file pone.0302641.s001.zip › minimal-dataset/image/0040_NI000_slice001.png]

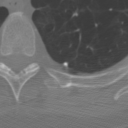

Supplement: S1 Dataset — (ZIP) [file pone.0302641.s001.zip › minimal-dataset/image/0041_NI002_slice001.png]

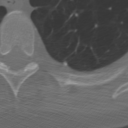

Supplement: S1 Dataset — (ZIP) [file pone.0302641.s001.zip › minimal-dataset/image/0041_NI002_slice002.png]

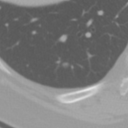

Supplement: S1 Dataset — (ZIP) [file pone.0302641.s001.zip › minimal-dataset/image/0042_NI000_slice000.png]

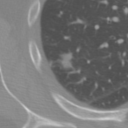

Supplement: S1 Dataset — (ZIP) [file pone.0302641.s001.zip › minimal-dataset/image/0043_NI002_slice001.png]

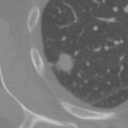

Supplement: S1 Dataset — (ZIP) [file pone.0302641.s001.zip › minimal-dataset/image/0043_NI002_slice002.png]

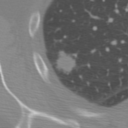

Supplement: S1 Dataset — (ZIP) [file pone.0302641.s001.zip › minimal-dataset/image/0043_NI002_slice003.png]

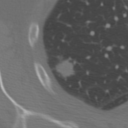

Supplement: S1 Dataset — (ZIP) [file pone.0302641.s001.zip › minimal-dataset/image/0043_NI002_slice004.png]

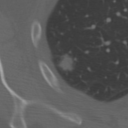

Supplement: S1 Dataset — (ZIP) [file pone.0302641.s001.zip › minimal-dataset/image/0043_NI002_slice005.png]

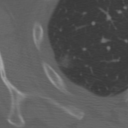

Supplement: S1 Dataset — (ZIP) [file pone.0302641.s001.zip › minimal-dataset/image/0043_NI002_slice006.png]

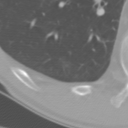

Supplement: S1 Dataset — (ZIP) [file pone.0302641.s001.zip › minimal-dataset/image/0044_NI000_slice000.png]

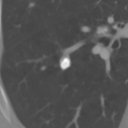

Supplement: S1 Dataset — (ZIP) [file pone.0302641.s001.zip › minimal-dataset/image/0044_NI001_slice001.png]

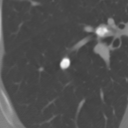

Supplement: S1 Dataset — (ZIP) [file pone.0302641.s001.zip › minimal-dataset/image/0044_NI001_slice002.png]

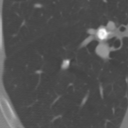

Supplement: S1 Dataset — (ZIP) [file pone.0302641.s001.zip › minimal-dataset/image/0044_NI001_slice003.png]

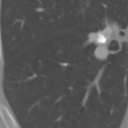

Supplement: S1 Dataset — (ZIP) [file pone.0302641.s001.zip › minimal-dataset/image/0044_NI001_slice004.png]

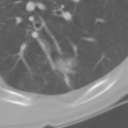

Supplement: S1 Dataset — (ZIP) [file pone.0302641.s001.zip › minimal-dataset/image/0044_NI002_slice001.png]

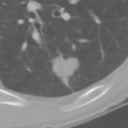

Supplement: S1 Dataset — (ZIP) [file pone.0302641.s001.zip › minimal-dataset/image/0044_NI002_slice002.png]
