# Supplementary figures and images for: Attention pyramid pooling network for artificial diagnosis on pulmonary nodules (part 1 of 2)
Source: PLoS One. 2024 May 16;19(5):e0302641. doi: 10.1371/journal.pone.0302641 (PMC11098435; doi:10.1371/journal.pone.0302641)

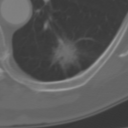

Supplement: S1 Dataset — (ZIP) [file pone.0302641.s001.zip › minimal-dataset/image/0001_NI000_slice001.png]

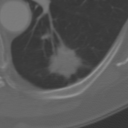

Supplement: S1 Dataset — (ZIP) [file pone.0302641.s001.zip › minimal-dataset/image/0001_NI000_slice002.png]

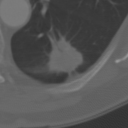

Supplement: S1 Dataset — (ZIP) [file pone.0302641.s001.zip › minimal-dataset/image/0001_NI000_slice003.png]

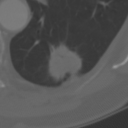

Supplement: S1 Dataset — (ZIP) [file pone.0302641.s001.zip › minimal-dataset/image/0001_NI000_slice004.png]

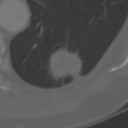

Supplement: S1 Dataset — (ZIP) [file pone.0302641.s001.zip › minimal-dataset/image/0001_NI000_slice005.png]

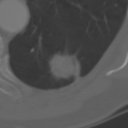

Supplement: S1 Dataset — (ZIP) [file pone.0302641.s001.zip › minimal-dataset/image/0001_NI000_slice006.png]

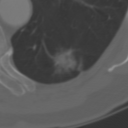

Supplement: S1 Dataset — (ZIP) [file pone.0302641.s001.zip › minimal-dataset/image/0001_NI000_slice007.png]

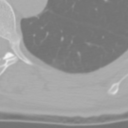

Supplement: S1 Dataset — (ZIP) [file pone.0302641.s001.zip › minimal-dataset/image/0002_NI000_slice000.png]

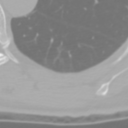

Supplement: S1 Dataset — (ZIP) [file pone.0302641.s001.zip › minimal-dataset/image/0002_NI000_slice001.png]

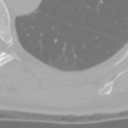

Supplement: S1 Dataset — (ZIP) [file pone.0302641.s001.zip › minimal-dataset/image/0002_NI000_slice002.png]

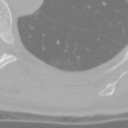

Supplement: S1 Dataset — (ZIP) [file pone.0302641.s001.zip › minimal-dataset/image/0002_NI000_slice003.png]

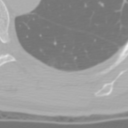

Supplement: S1 Dataset — (ZIP) [file pone.0302641.s001.zip › minimal-dataset/image/0002_NI000_slice004.png]

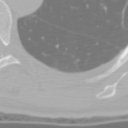

Supplement: S1 Dataset — (ZIP) [file pone.0302641.s001.zip › minimal-dataset/image/0002_NI000_slice005.png]

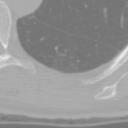

Supplement: S1 Dataset — (ZIP) [file pone.0302641.s001.zip › minimal-dataset/image/0002_NI000_slice006.png]

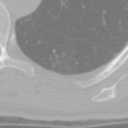

Supplement: S1 Dataset — (ZIP) [file pone.0302641.s001.zip › minimal-dataset/image/0002_NI000_slice007.png]

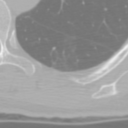

Supplement: S1 Dataset — (ZIP) [file pone.0302641.s001.zip › minimal-dataset/image/0002_NI000_slice008.png]

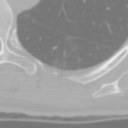

Supplement: S1 Dataset — (ZIP) [file pone.0302641.s001.zip › minimal-dataset/image/0002_NI000_slice009.png]

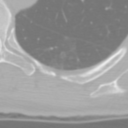

Supplement: S1 Dataset — (ZIP) [file pone.0302641.s001.zip › minimal-dataset/image/0002_NI000_slice010.png]

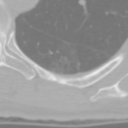

Supplement: S1 Dataset — (ZIP) [file pone.0302641.s001.zip › minimal-dataset/image/0002_NI000_slice011.png]

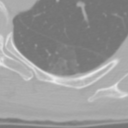

Supplement: S1 Dataset — (ZIP) [file pone.0302641.s001.zip › minimal-dataset/image/0002_NI000_slice012.png]

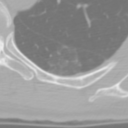

Supplement: S1 Dataset — (ZIP) [file pone.0302641.s001.zip › minimal-dataset/image/0002_NI000_slice013.png]

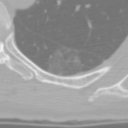

Supplement: S1 Dataset — (ZIP) [file pone.0302641.s001.zip › minimal-dataset/image/0002_NI000_slice014.png]

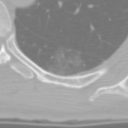

Supplement: S1 Dataset — (ZIP) [file pone.0302641.s001.zip › minimal-dataset/image/0002_NI000_slice015.png]

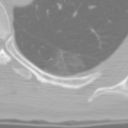

Supplement: S1 Dataset — (ZIP) [file pone.0302641.s001.zip › minimal-dataset/image/0002_NI000_slice016.png]

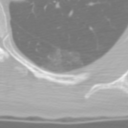

Supplement: S1 Dataset — (ZIP) [file pone.0302641.s001.zip › minimal-dataset/image/0002_NI000_slice017.png]

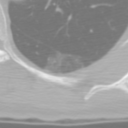

Supplement: S1 Dataset — (ZIP) [file pone.0302641.s001.zip › minimal-dataset/image/0002_NI000_slice018.png]

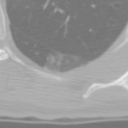

Supplement: S1 Dataset — (ZIP) [file pone.0302641.s001.zip › minimal-dataset/image/0002_NI000_slice019.png]

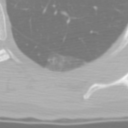

Supplement: S1 Dataset — (ZIP) [file pone.0302641.s001.zip › minimal-dataset/image/0002_NI000_slice020.png]

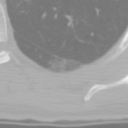

Supplement: S1 Dataset — (ZIP) [file pone.0302641.s001.zip › minimal-dataset/image/0002_NI000_slice021.png]

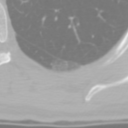

Supplement: S1 Dataset — (ZIP) [file pone.0302641.s001.zip › minimal-dataset/image/0002_NI000_slice022.png]

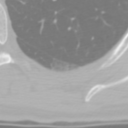

Supplement: S1 Dataset — (ZIP) [file pone.0302641.s001.zip › minimal-dataset/image/0002_NI000_slice023.png]

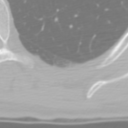

Supplement: S1 Dataset — (ZIP) [file pone.0302641.s001.zip › minimal-dataset/image/0002_NI000_slice024.png]

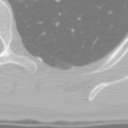

Supplement: S1 Dataset — (ZIP) [file pone.0302641.s001.zip › minimal-dataset/image/0002_NI000_slice025.png]

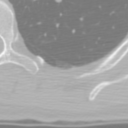

Supplement: S1 Dataset — (ZIP) [file pone.0302641.s001.zip › minimal-dataset/image/0002_NI000_slice026.png]

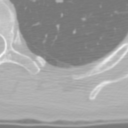

Supplement: S1 Dataset — (ZIP) [file pone.0302641.s001.zip › minimal-dataset/image/0002_NI000_slice027.png]

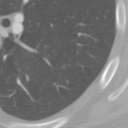

Supplement: S1 Dataset — (ZIP) [file pone.0302641.s001.zip › minimal-dataset/image/0003_NI000_slice000.png]

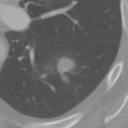

Supplement: S1 Dataset — (ZIP) [file pone.0302641.s001.zip › minimal-dataset/image/0003_NI001_slice003.png]

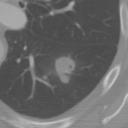

Supplement: S1 Dataset — (ZIP) [file pone.0302641.s001.zip › minimal-dataset/image/0003_NI001_slice004.png]

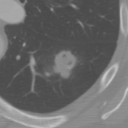

Supplement: S1 Dataset — (ZIP) [file pone.0302641.s001.zip › minimal-dataset/image/0003_NI001_slice005.png]

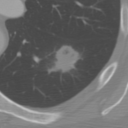

Supplement: S1 Dataset — (ZIP) [file pone.0302641.s001.zip › minimal-dataset/image/0003_NI001_slice006.png]

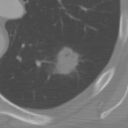

Supplement: S1 Dataset — (ZIP) [file pone.0302641.s001.zip › minimal-dataset/image/0003_NI001_slice007.png]

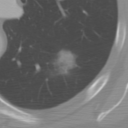

Supplement: S1 Dataset — (ZIP) [file pone.0302641.s001.zip › minimal-dataset/image/0003_NI001_slice008.png]

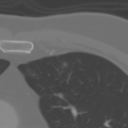

Supplement: S1 Dataset — (ZIP) [file pone.0302641.s001.zip › minimal-dataset/image/0003_NI002_slice000.png]

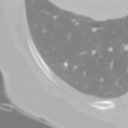

Supplement: S1 Dataset — (ZIP) [file pone.0302641.s001.zip › minimal-dataset/image/0004_NI000_slice001.png]

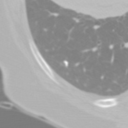

Supplement: S1 Dataset — (ZIP) [file pone.0302641.s001.zip › minimal-dataset/image/0004_NI000_slice002.png]

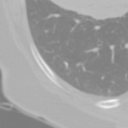

Supplement: S1 Dataset — (ZIP) [file pone.0302641.s001.zip › minimal-dataset/image/0004_NI000_slice003.png]

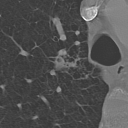

Supplement: S1 Dataset — (ZIP) [file pone.0302641.s001.zip › minimal-dataset/image/0007_NI000_slice002.png]

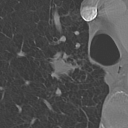

Supplement: S1 Dataset — (ZIP) [file pone.0302641.s001.zip › minimal-dataset/image/0007_NI000_slice003.png]

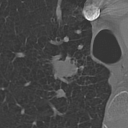

Supplement: S1 Dataset — (ZIP) [file pone.0302641.s001.zip › minimal-dataset/image/0007_NI000_slice004.png]

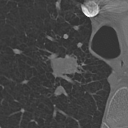

Supplement: S1 Dataset — (ZIP) [file pone.0302641.s001.zip › minimal-dataset/image/0007_NI000_slice005.png]

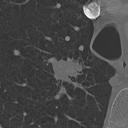

Supplement: S1 Dataset — (ZIP) [file pone.0302641.s001.zip › minimal-dataset/image/0007_NI000_slice006.png]

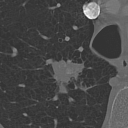

Supplement: S1 Dataset — (ZIP) [file pone.0302641.s001.zip › minimal-dataset/image/0007_NI000_slice007.png]

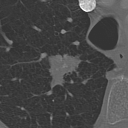

Supplement: S1 Dataset — (ZIP) [file pone.0302641.s001.zip › minimal-dataset/image/0007_NI000_slice008.png]

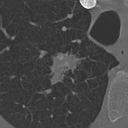

Supplement: S1 Dataset — (ZIP) [file pone.0302641.s001.zip › minimal-dataset/image/0007_NI000_slice009.png]

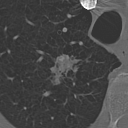

Supplement: S1 Dataset — (ZIP) [file pone.0302641.s001.zip › minimal-dataset/image/0007_NI000_slice010.png]

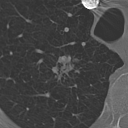

Supplement: S1 Dataset — (ZIP) [file pone.0302641.s001.zip › minimal-dataset/image/0007_NI000_slice011.png]

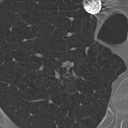

Supplement: S1 Dataset — (ZIP) [file pone.0302641.s001.zip › minimal-dataset/image/0007_NI000_slice012.png]

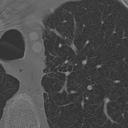

Supplement: S1 Dataset — (ZIP) [file pone.0302641.s001.zip › minimal-dataset/image/0007_NI001_slice000.png]

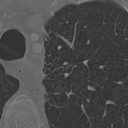

Supplement: S1 Dataset — (ZIP) [file pone.0302641.s001.zip › minimal-dataset/image/0007_NI001_slice001.png]

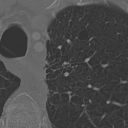

Supplement: S1 Dataset — (ZIP) [file pone.0302641.s001.zip › minimal-dataset/image/0007_NI001_slice002.png]

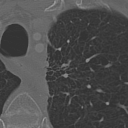

Supplement: S1 Dataset — (ZIP) [file pone.0302641.s001.zip › minimal-dataset/image/0007_NI001_slice003.png]

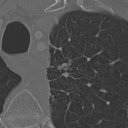

Supplement: S1 Dataset — (ZIP) [file pone.0302641.s001.zip › minimal-dataset/image/0007_NI001_slice004.png]

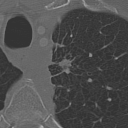

Supplement: S1 Dataset — (ZIP) [file pone.0302641.s001.zip › minimal-dataset/image/0007_NI001_slice005.png]

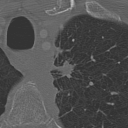

Supplement: S1 Dataset — (ZIP) [file pone.0302641.s001.zip › minimal-dataset/image/0007_NI001_slice006.png]

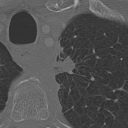

Supplement: S1 Dataset — (ZIP) [file pone.0302641.s001.zip › minimal-dataset/image/0007_NI001_slice007.png]

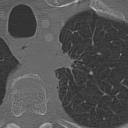

Supplement: S1 Dataset — (ZIP) [file pone.0302641.s001.zip › minimal-dataset/image/0007_NI001_slice008.png]

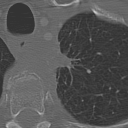

Supplement: S1 Dataset — (ZIP) [file pone.0302641.s001.zip › minimal-dataset/image/0007_NI001_slice009.png]

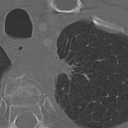

Supplement: S1 Dataset — (ZIP) [file pone.0302641.s001.zip › minimal-dataset/image/0007_NI001_slice010.png]

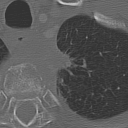

Supplement: S1 Dataset — (ZIP) [file pone.0302641.s001.zip › minimal-dataset/image/0007_NI001_slice011.png]

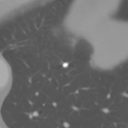

Supplement: S1 Dataset — (ZIP) [file pone.0302641.s001.zip › minimal-dataset/image/0011_NI001_slice001.png]

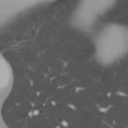

Supplement: S1 Dataset — (ZIP) [file pone.0302641.s001.zip › minimal-dataset/image/0011_NI001_slice002.png]

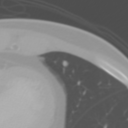

Supplement: S1 Dataset — (ZIP) [file pone.0302641.s001.zip › minimal-dataset/image/0011_NI002_slice001.png]

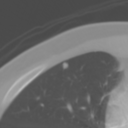

Supplement: S1 Dataset — (ZIP) [file pone.0302641.s001.zip › minimal-dataset/image/0011_NI004_slice001.png]

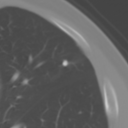

Supplement: S1 Dataset — (ZIP) [file pone.0302641.s001.zip › minimal-dataset/image/0011_NI005_slice001.png]

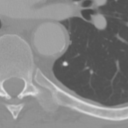

Supplement: S1 Dataset — (ZIP) [file pone.0302641.s001.zip › minimal-dataset/image/0011_NI006_slice001.png]

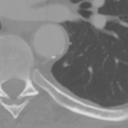

Supplement: S1 Dataset — (ZIP) [file pone.0302641.s001.zip › minimal-dataset/image/0011_NI006_slice002.png]

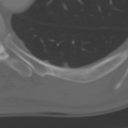

Supplement: S1 Dataset — (ZIP) [file pone.0302641.s001.zip › minimal-dataset/image/0011_NI007_slice000.png]

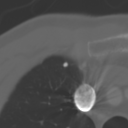

Supplement: S1 Dataset — (ZIP) [file pone.0302641.s001.zip › minimal-dataset/image/0011_NI008_slice001.png]

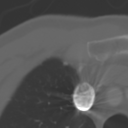

Supplement: S1 Dataset — (ZIP) [file pone.0302641.s001.zip › minimal-dataset/image/0011_NI008_slice002.png]

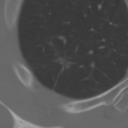

Supplement: S1 Dataset — (ZIP) [file pone.0302641.s001.zip › minimal-dataset/image/0011_NI009_slice000.png]

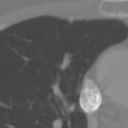

Supplement: S1 Dataset — (ZIP) [file pone.0302641.s001.zip › minimal-dataset/image/0013_NI002_slice001.png]

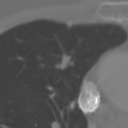

Supplement: S1 Dataset — (ZIP) [file pone.0302641.s001.zip › minimal-dataset/image/0013_NI002_slice002.png]

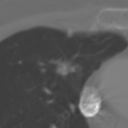

Supplement: S1 Dataset — (ZIP) [file pone.0302641.s001.zip › minimal-dataset/image/0013_NI002_slice003.png]

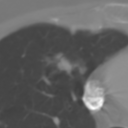

Supplement: S1 Dataset — (ZIP) [file pone.0302641.s001.zip › minimal-dataset/image/0013_NI002_slice004.png]

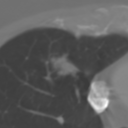

Supplement: S1 Dataset — (ZIP) [file pone.0302641.s001.zip › minimal-dataset/image/0013_NI002_slice005.png]

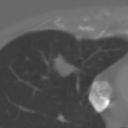

Supplement: S1 Dataset — (ZIP) [file pone.0302641.s001.zip › minimal-dataset/image/0013_NI002_slice006.png]

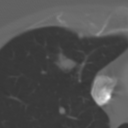

Supplement: S1 Dataset — (ZIP) [file pone.0302641.s001.zip › minimal-dataset/image/0013_NI002_slice007.png]

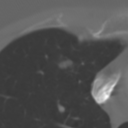

Supplement: S1 Dataset — (ZIP) [file pone.0302641.s001.zip › minimal-dataset/image/0013_NI002_slice008.png]

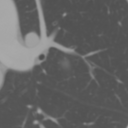

Supplement: S1 Dataset — (ZIP) [file pone.0302641.s001.zip › minimal-dataset/image/0014_NI000_slice000.png]

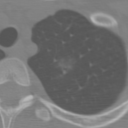

Supplement: S1 Dataset — (ZIP) [file pone.0302641.s001.zip › minimal-dataset/image/0015_NI000_slice003.png]

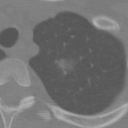

Supplement: S1 Dataset — (ZIP) [file pone.0302641.s001.zip › minimal-dataset/image/0015_NI000_slice004.png]

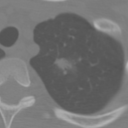

Supplement: S1 Dataset — (ZIP) [file pone.0302641.s001.zip › minimal-dataset/image/0015_NI000_slice005.png]

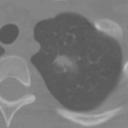

Supplement: S1 Dataset — (ZIP) [file pone.0302641.s001.zip › minimal-dataset/image/0015_NI000_slice006.png]

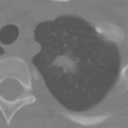

Supplement: S1 Dataset — (ZIP) [file pone.0302641.s001.zip › minimal-dataset/image/0015_NI000_slice007.png]

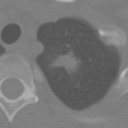

Supplement: S1 Dataset — (ZIP) [file pone.0302641.s001.zip › minimal-dataset/image/0015_NI000_slice008.png]

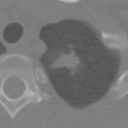

Supplement: S1 Dataset — (ZIP) [file pone.0302641.s001.zip › minimal-dataset/image/0015_NI000_slice009.png]

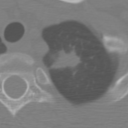

Supplement: S1 Dataset — (ZIP) [file pone.0302641.s001.zip › minimal-dataset/image/0015_NI000_slice010.png]

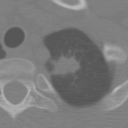

Supplement: S1 Dataset — (ZIP) [file pone.0302641.s001.zip › minimal-dataset/image/0015_NI000_slice011.png]

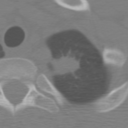

Supplement: S1 Dataset — (ZIP) [file pone.0302641.s001.zip › minimal-dataset/image/0015_NI000_slice012.png]

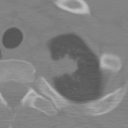

Supplement: S1 Dataset — (ZIP) [file pone.0302641.s001.zip › minimal-dataset/image/0015_NI000_slice013.png]
